# Lab-on-Chip, Surface-Enhanced Raman Analysis by Aerosol Jet Printing and Roll-to-Roll Hot Embossing

**DOI:** 10.3390/s17102401

**Published:** 2017-10-20

**Authors:** Anne Habermehl, Noah Strobel, Ralph Eckstein, Nico Bolse, Adrian Mertens, Gerardo Hernandez-Sosa, Carsten Eschenbaum, Uli Lemmer

**Affiliations:** 1 Light Technology Institute, Karlsruhe Institute of Technology (KIT), Engesserstraße 13, 76131 Karlsruhe, Germany; noah.strobel@kit.edu (N.S.); ralph.eckstein@kit.edu (R.E.); nico.bolse@kit.edu (N.B.); adrian.mertens@kit.edu (A.M.); gerardo.sosa@kit.edu (G.H.-S.); carsten.eschenbaum@kit.edu (C.E.); uli.lemmer@kit.edu (U.L.); 2 InnovationLab GmbH, Speyerer Straße 4, 69115 Heidelberg, Germany; 3 Institute of Microstructure Technology, Karlsruhe Institute of Technology (KIT), Hermann-von-Helmholtz-Platz 1, 76344 Eggenstein-Leopoldshafen, Germany

**Keywords:** surface-enhanced Raman spectroscopy, aerosol jet printing, roll-to-roll, microfluidics, low-cost, bioanalysis

## Abstract

Surface-enhanced Raman spectroscopy (SERS) combines the high specificity of Raman scattering with high sensitivity due to an enhancement of the electromagnetic field by metallic nanostructures. However, the tyical fabrication methods of SERS substrates suffer from low throughput and therefore high costs. Furthermore, point-of-care applications require the investigation of liquid solutions and thus the integration of the SERS substrate in a microfluidic chip. We present a roll-to-roll fabrication approach for microfluidics with integrated, highly efficient, surface-enhanced Raman scattering structures. Microfluidic channels are formed using roll-to-roll hot embossing in polystyrene foil. Aerosol jet printing of a gold nanoparticle ink is utilized to manufacture highly efficient, homogeneous, and reproducible SERS structures. The modified channels are sealed with a solvent-free, roll-to-roll, thermal bonding process. In continuous flow measurements, these chips overcome time-consuming incubation protocols and the poor reproducibility of SERS experiments often caused by inhomogeneous drying of the analyte. In the present study, we explore the influence of the printing process on the homogeneity and the enhancement of the SERS structures. The feasibility of aerosol-jet-modified microfluidic channels for highly sensitive SERS detection is demonstrated by using solutions with different concentrations of Rhodamine 6G and adenosine. The printed areas provide homogeneous enhancement factors of ~4 × 10^6^. Our work shows a way towards the low-cost production of tailor-made, SERS-enabled, label-free, lab-on- chip systems for bioanalysis.

## 1. Introduction

In metallic nanostructures, localized surface-plasmon resonances lead to an enhancement of the Raman scattering signal. Therefore, surface-enhanced Raman spectroscopy (SERS) combines the high specificity of Raman spectroscopy with high sensitivity. SERS is an ideal detection method for the quantitative molecular analysis of small sample volumes of aqueous solutions in microfluidics [1,2,3,4,5,6,7,8]. The SERS analysis is facilitated and accelerated when the analyte is not drop-casted or incubated on the SERS substrates, but continuously flowing through a microfluidic chip with integrated SERS detection [9]. Thus, the combination of SERS and microfluidics is well suited for field applications. Here, the cost-efficiency and the compatibility of the fabrication processes of the microfluidics and enhancing structures have to be ensured. 

High Raman enhancement factors can be achieved by a variety of metallic nanostructures, such as rough metal surfaces, periodic metal nanostructures or a distribution of metal nanoparticles. For the realization, electron beam lithography, nanoimprint lithography, nanosphere lithography, etching, or simple spincoating have been used [10,11,12,13]. However, those fabrication methods are often complex, time-consuming or not easily combinable with microfluidics and thus not suitable for the mass production of integrated microfluidic SERS chips. In the context of low-cost fabrication, printing technologies are highly attractive for the fabrication of SERS substrates based on the nanoparticle approach. Besides the ink formulation and preparation, the printing process itself plays an important role. Several printing processes, e.g., screen-printing and gravure printing, have already been investigated for the fabrication of low cost SERS substrates [14,15,16,17]. Digital printing technologies additionally allow for a well-defined and contact-free deposition of the ink, which is essential for a modification of the microchannels without extensive alignment. Ink-jet printing is a very versatile digital printing technology [18,19,20,21]. It requires, however, careful tuning of the ink formulation and, furthermore, relatively large amounts of solvents, which are deposited on the substrates and dissolve many polymeric materials. 

In this contribution, we demonstrate aerosol jet printing (AJP) as an advantageous method that offers a wide choice of substrates as the amount of solvents in the aerosol can be controlled and therefore also substrates that are not stable to solvents can be used. This applies, e.g., to the case of polystyrene. Furthermore, the better resolution of AJP compared to ink-jet printing renders this method an ideal candidate for the modification of microfluidics [22,23]. 

Microfluidic chips made of glass or polydimethylsiloxane (PDMS) or a combination of both materials are widely used. Glass microfluidics can only be fabricated using low-throughput technologies, e.g., etching technology or machining tools [24,25,26,27,28], but they offer the advantage of a low Raman background signal. PDMS microfluidics on the other hand are more easily fabricated using soft lithography [2,29,30]. A high-throughput fabrication process is nevertheless not possible with PDMS and, additionally, the background Raman signal of PDMS chips, their swelling and the absorption of analyte molecules, are inherent material properties and prevent analytical solutions. Thus, for a low-cost fabrication of SERS chips both materials present disadvantages. As an alternative approach, thermoplastic polymer microfluidic devices fabricated by hot embossing have been investigated for chemical and biological applications [31]. In order to further decrease the production cost, the widely used flat-to-flat embossing process can be replaced by a roll-to-roll (R2R) process. R2R fabrication of polymeric microfluidic chips has already been investigated for several applications, all using solvents or adhesive layers for bonding [32,33,34,35,36]. These additional layers may cause background signals during the Raman measurements and their deposition can be problematic for the SERS structures themselves.

In this paper, we present an easy and reliable three-step fabrication process of microfluidic chips for surface-enhanced Raman analysis. First, R2R hot embossing is employed for the fabrication of microfluidic channels in polystyrene (PS) using high temperatures and pressure stable masters made by soft-lithography and low-cost materials. Second, AJP of a nanoparticle gold (Au) ink is used for the integration of the SERS structures into the channels. Third, the chips are finally sealed by simple thermal R2R bonding. As the reproducibility of SERS chips and the uniformity of the enhancement over the SERS area are crucial for any application, we investigate the influence of several printing parameters on the SERS performance of the chips using rhodamine 6G (Rh6G) and adenosine as analytes and determine the processing parameters resulting in high-performance lab-on-chip (LoC)-SERS systems.

## 2. Materials and Methods

### 2.1. Master Fabrication for Roll-to-Roll Hot Embossing

A master that is stable at high temperatures and pressures, flexible, and preferentially low-cost, is required for R2R hot embossing. Thus, we fabricated an epoxy master on steel foil to be attached to our magnetic embossing cylinder using low-cost materials and soft-lithography. The epoxy master was superior in terms of flexibility and stability vs. pressure and heat as compared to SU-8. In comparison to nickel shims, which are often used in hot embossing, the costs are extremely low and the silicon master can be used multiple times. In a first step, the inverse structure of the microfluidic channel was fabricated via UV-lithography in SU-8 2025 (micro resist technology GmbH, Berlin, Deutschland) on 4’’ silicon wafers. The channel dimensions were 4 mm in length, 400 µm in width and 57 µm in height. The SU-8 structures were replicated in the two-component PDMS Sylgard 184 (Dow Corning GmbH, Wiesbaden, Germany). For this purpose, the PDMS base type and curing agent were mixed in a ratio 10:1 and carefully poured over the SU-8 structures. The PDMS was cured at 100 °C for 20 min. The resulting PDMS stamp was subsequently replicated in the two-component epoxy MP Advanced (R&G Faserverbundwerkstoffe GmbH, Waldenbuch, Germany) on top of the steel foil (Mercateo AG, München, Germany). This procedure allows fast replication from a rigid to a flexible substrate. It should be mentioned that the lifetime of such an epoxy master cannot compete with a nickel master at high embossing pressures. The replication process from the rigid silicon towards the flexible master is depicted in Figure S1 in the Supplementary Materials.

### 2.2. Fabrication of SERS Chips

**Figure 1:**
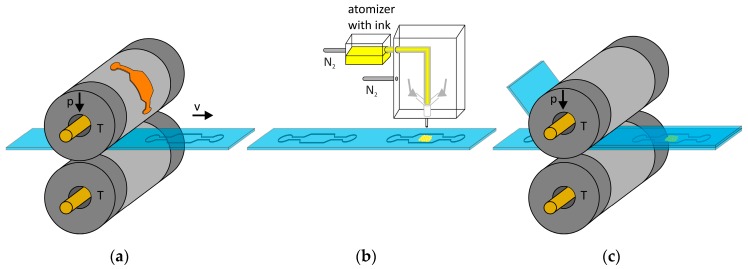
Fabrication process of optofluidic chips for surface-enhanced Raman spectroscopy (SERS). roll-to-roll (R2R) hot embossing of polystyrene (PS) foil (**a**), followed by modification with aerosol jet printed Au nanoparticles (**b**) and sealing of the chip with a PS cover foil by R2R bonding (**c**).

The roll-to-roll fabrication process of the microfluidic chips and its modification using AJP is shown in Figure 1 and consists of three fully R2R-compatible steps. 

#### 2.2.1. Roll-to-Roll Hot Embossing of the Microfluidic Channel

For the transfer of the master structures in the PS foil (ergo.win, Norflex GmbH, Ingolstadt, Germany), we implemented a custom-made R2R hot embossing scheme (Figure 1a). By heating two cylinders above the glass transition temperature of PS and applying a pressing force on the upper cylinder, the structures were embossed in the polymer foil that was guided between the two cylinders. 

#### 2.2.2. Aerosol Jet Printing

An aerosol jet printer (AJ 300, Optomec, Albuquerque, NM, USA) was used to deposit ~50 µm wide lines of Au nanoparticles as enhancing structures for SERS directly into the microfluidic channel. A nanoparticle Au ink with particle sizes of 80 nm was printed (Au-LT-20 by Fraunhofer IKTS, Dresden, Germany). The working principle of AJP is schematically shown in Figure 1b. The Au ink was atomized by ultrasonication and the generated sub-micron droplets were then carried to the nozzle head by a nitrogen carrier gas flow. The material stream was focused within the nozzle head by a sheath gas flow. The resulting collimated co-axial stream of aerosol and sheath gas was guided through a ceramic nozzle tip onto the substrate at a distance of 5 to 10 mm underneath. Furthermore, a mechanical shutter arm allowed on-demand deposition of the Au nanoparticles. In order to investigate the influence of variations in the printing process we printed lines at two tube temperatures of 20 °C and 60 °C, respectively, and with printing cycles per line from 5 to 12 at a velocity of 2 mm/s. The lines had a spacing of 500 µm. We investigated several post printing processes such as oxygen plasma treatment, vacuum drying and rinsing to remove residuals. The modification of the channels enabled easy bonding after the functionalization as a complex alignment was unnecessary.

#### 2.2.3. Roll-to-Roll Thermal Bonding

In order to allow for the SERS analysis of liquid analytes, the chips had to be sealed after the AJP process. This was accomplished by using the R2R setup again to thermally bond the PS microfluidics with a PS cover foil (see Figure 1c). For this purpose, the previously structured PS foil with printed nanoparticles was covered with a second PS foil and again guided in between the two heated cylinders. This led to a permanent bonding at the interface of the two foils.

### 2.3. SERS Measurements

All Raman and SERS measurements using our microfluidic chip were obtained from solutions at a flow rate of 30 µL/min, controlled by a syringe pump (LA 30, Landgraf Laborsysteme HLL GmbH, Langenhagen, Germany). Analyte solutions were stocked in a syringe (5 mL Injekt, B. Braun Melsungen AG, Melsungen, Germany) and delivered to our SERS chips mounted on a 3D-printed sample holder allowing for simple fluidic contact with one inlet and one outlet. The measurements were performed at room temperature using a 632.8 nm helium-neon laser for excitation. The light was filtered by a clean-up filter (MaxLine laser clean-up 633, Semrock, Rochester, NY, USA) and a dichroic mirror (RazorEdge Dichroic 633 RU, Semrock, Rochester, NY, USA). Finally, a 40× objective (NA 0.6) focused the light through the microfluidic channels on the Au nanoparticles. The diameter of the laser spot was ~6 µm and the excitation power was 0.9 mW, if not indicated differently. The back-scattered Raman signals were detected by a spectrograph (Acton Spectra Pro 2500i, Princeton Instruments, Trenton, NJ, USA) equipped with an electron-multiplying charge-coupled device (EMCCD) camera (iXon, Andor, Belfast, UK) following a longpass filter (RazorEdge LP Edge Filter 633 RU, AHF Analysetechnik, Tübingen, Germany). To demonstrate the capability of SERS detection in microfluidic channels, Rhodamine 6G (Radiant Dyes Laser & Accessories GmbH, Wermelskirchen, Germany) and adenosine (Sigma-Aldrich Chemie GmbH, Taufkirchen, Germany) were used as analytes. Concentration-dependent measurements were carried out starting with the lowest concentration, followed by a stepwise increase of the concentration of the analyte by one order of magnitude. The corresponding SERS spectra were recorded after 5 min allowing the concentration and thus the signal to reach a stabilized equilibrium.

## 3. Results

### 3.1. Optimization of the Microfluidic Chip

**Figure 2:**
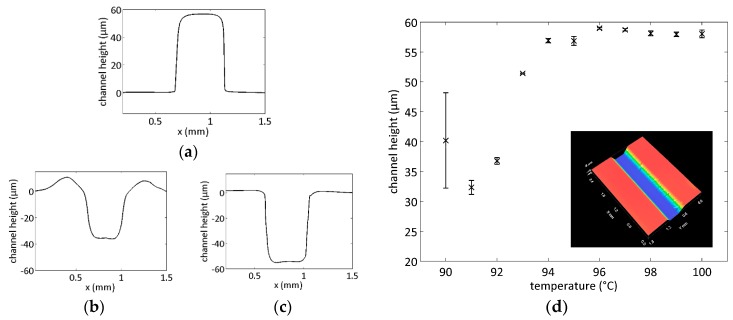
Height profile of the epoxy structure (**a**) and the embossed PS channels using cylinder temperatures of 91 °C (**b**) and 96 °C (**c**). The dependence of the channel depth on the cylinders’ temperature is given in (**d**), the white light interferometer scan of 1.8 × 2.4 mm^2^ of the 96 °C-channel in the inset shows the good regularity of the embossed channels.

The microfluidic chip served as a platform for the integration of SERS structures and for the delivery of the liquid analyte. The quality of the replicated structure depends on the embossing temperature, the applied pressure and the foil velocity. The most critical parameter for the chip fabrication in PS was the temperature. The surface profile of the epoxy master attached to one cylinder shows a height of ~58 µm (Figure 2a). For low temperatures, the structure transfer was not complete (Figure 2b). The master squeezed the material to the sides, which lead to elevations next of the channel. It was observed that the master was not penetrating at its full height. In the temperature range of 94 to 99 °C the channel was transferred completely (Figure 2c). Temperatures higher than 99 °C led to a sticking of the polymer foil to the epoxy master, which increased the web tension and hindered an R2R process. Figure 2d depicts the dependence of the channel depth on the temperature of the cylinders with a glass transition of PS between 90 °C and 94 °C and a large embossing window from 94 °C to 99 °C [37]. The white light interferometer scan in the inset of Figure 2d proves the good homogeneity of the embossed channel. The applied additional pressure was one bar and the velocity was 12 cm/min. Higher pressure or velocity did not lead to better embossing results. The embossed channels were sealed with a second PS foil using the same R2R setup with unstructured cylinders. We investigated the bonding process with the channels embossed at 96 °C. In this step, the pressure was provided by the bare weight of the upper cylinder. Higher pressure led to a partial closing of the microfluidic channels. For temperatures lower than 92 °C, the channels were leaky or even completely delaminating. A bonding temperature higher than 94 °C resulted again in blocked channels. We achieved good sealing results with T = 93 °C at a velocity of 15 cm/min. In order to evaluate the strength of this bonding, we mounted the chip in the chip holder and raised the flow rate of water through the microfluidic channel. These PS microfluidic chips burst when the flow rate exceeded 1000 µL/min. The Raman measurements were conducted at a through flow of 30 µL/min and all the used chips consequently did not show any degradation under those conditions. We thus fabricated microfluidic channels with full structure transfer with our custom-made R2R hot embossing setup and subsequently thermally bonded them without any additional bonding layers. Herewith, we obtained fully R2R-fabricated microfluidic chips. 

### 3.2. Characterization of Aerosol-Jet-Printed SERS Structures

An important aspect in this work is the influence of the aerosol jet printing parameters on the homogeneity and the enhancement of the SERS structures in order to prove the reliability of the fabrication process. This is especially essential for field applications where easy plug-and-play measurements should be guaranteed. 

**Figure 3:**
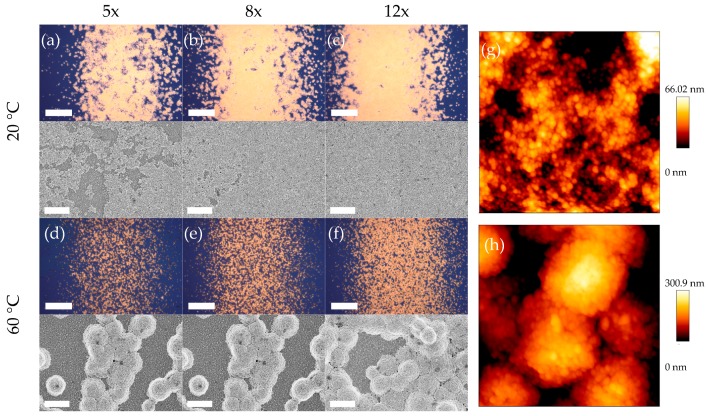
Light microscope images (scale bar 20 µm), scanning electron micrographs (scale bar 1 µm) and atomic force micrographs (2 × 2 µm^2^) of the nanoparticles printed at 20 °C (**a**–**c**,**g**) and at 60 °C (**d**–**f**,**h**) with different printing cycles.

Figure 3 depicts light microscopic images, scanning electron micrographs, and atomic force micrographs of the aerosol-jet-printed Au lines for different tube temperatures and printing cycles. The density of the nanoparticle layers was increased by raising the number of printing cycles from 5 to 12. By doing so, homogeneously coated and pinhole free lines could be achieved for a tube temperature of 20 °C. For a tube temperature of 60 °C some pinholes remained, even with high numbers of printing cycles. The influence of the tube temperature becomes more evident in the SEM and AFM images. While 20 °C resulted in a homogeneous and smooth surface with a roughness of only R_q_ = 15.0 nm, a temperature of 60 °C led to the formation of agglomerates in the range of several microns. The agglomeration most likely happened during the transport of the aerosol to the substrate stemming from the enhanced evaporation rate of the solvent at 60 °C compared to a tube temperature of 20 °C. 

After the printing process, some residues remained on the Au nanoparticle layers which could be solvents or organic stabilizers of the ink as EDX measurements showed a high carbon content. Typically, printing processes are followed by temperature treatments. Due to the use of PS as a substrate material, an extensive heating of the printed layers was not possible. Oxygen plasma treatment, vacuum drying as well as rinsing with water proved to be effective methods to remove said residues. However, oxygen plasma led to a fusion of the Au particles, resulting in a decreasing SERS signal, as shown in Figure S4. Additionally, the plasma treatment affects the PS surface and subsequently prevented successful bonding after the printing process. The drawback of the vacuum drying process was its time expenditure. For these reasons, water rinsing was integrated in the measurement protocol. The adhesion of the nanoparticle layers to the PS channel was stable. A dissolution of the nanoparticles has not been observed.

The suitability of a helium–neon laser as an excitation source was proved by dark-field scattering measurements of SERS substrates printed with 20 °C and 60 °C tube temperature and printing cycles of twelve showing a maximum around 630 nm. The spectra are given in Figure S5.

**Figure 4:**
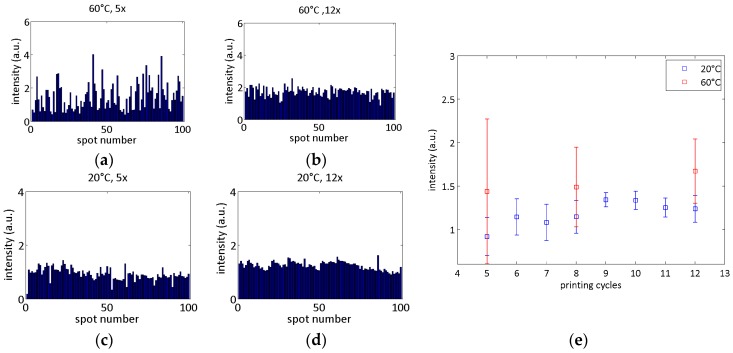
For SERS regularity measurements on printed nanoparticles, 10 µM Rh6G solution was used as analyte. Spectra were taken at 100 spots on an area of 20 × 20 µm^2^ with 10 s integration time. The Raman peak at 610 cm^−1^ was evaluated for comparison. On the left the results for 60 °C (**a**,**b**) and 20 °C (**c**,**d**) are exemplarily shown for 5 (**a**,**c**) and 12 (**b**,**d**) printing cycles. (**e**) shows the mean values and standard deviations for SERS measurements on samples fabricated with different printing cycles and tube temperatures in the overview.

The variation of the enhancement over the SERS area was investigated by recording the SERS spectra of a 10 µM solution of Rh6G at 100 spots in a 20 × 20 µm^2^ area on each line printed with two tube temperatures, printing cycles varying from 5 to 12, and evaluation of the intensity of the 610 cm^−1^ peak. The maximum signal of the 60 °C-chips was higher (see Figure 4). 

This can be explained on the one hand by the increased roughness of the printed lines (see Figure 3) which led to a larger surface and subsequently to more contributing molecules. On the other hand, the dark-field scattering spectra show a more pronounced peak for the 60 °C samples, which also explains the higher signal obtained with these samples. As shown in Figure 4, the uniformity of the Raman signal increased with an increased number of printing cycles and therefore with the increasing density of nanoparticles for both tube temperatures. At 20 °C tube temperature, a saturation of the mean value and its standard deviation in an acceptable range could be observed from nine to 12 printing cycles (see Figure 4e). As a relatively large processing window can be stated reliable fabrication should also be guaranteed for slight changes in the fabrication process or the substrate material. 

### 3.3. Quantitative SERS Analysis

**Figure 5:**
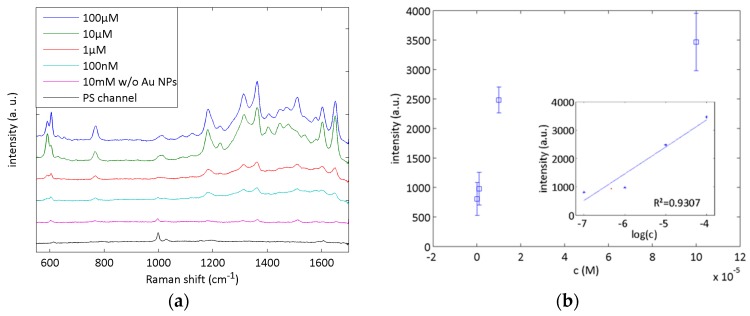
Microfluidic SERS analysis with Rh6G. Spectra of Rh6G solution at concentrations of 100 nM to 10 mM (**a**) were recorded with an acquisition time of 10 s and were averaged over 20 randomly chosen spots. The Raman peak at 780 cm^−1^ was evaluated for quantitative analysis (**b**), showing a good linear relationship between intensity and logarithmic concentration as shown in the inset.

After the qualitative evaluation of the microfluidic SERS chips, SERS measurement of Rh6G solutions with concentrations between 100 nM and 10 mM were conducted with the 20 °C chips. The spectra shown in Figure 5a were recorded with an acquisition time of 10 s and were averaged over 20 randomly chosen spots. The spectra are shifted vertically for better visibility. It can be seen that the SERS signal increased with an increasing analyte concentration. The Rh6G solution with a concentration of 10 mM was measured as a reference signal with an increased excitation power of 2.6 mW. Despite our measurement configuration, where the laser is focused through the channel onto the Au nanoparticles, the background spectrum of the microfluidic chip did not superimpose the SERS signals. Only the PS peak at 998 cm^−1^ can be observed in all spectra. The intensity of the Rh6G Raman peak at 766 cm^−1^ was measured (see Figure 5b) and a linear relationship in the logarithmic plot in the inset was observed as expected [19]. These results indicate the suitability of the aerosol-jet-printed SERS chips for quantitative SERS analysis. A theoretical detection limit for the Rh6G solution with 0.9 mW excitation power and 10 s integration time was calculated to 28.9 nM using the fit equation I_766_ = 947.9 × log(c) + 7147 and the intersection with I_766_ = 0. Given the standard deviation of the measurements, the real detection limit will be higher. 

Based on these results, the SERS enhancement factor (EF) can be calculated following the widely used equation *EF = (I_SERS_/I_Ref_)* × *(N_Ref_/N_SERS_)*, where *I_SERS_* is the signal intensity in presence of the Au nanoparticles and *I_Ref_* is the Raman intensity in absence of enhancement structures. The number of molecules contributing to the SERS signal can be estimated using *N_SERS_ = c_SERS_N_A_A_laser_h_Au_f_Rh6G_* with the Avogadro’s number *N_A_.* It depends on the analyte concentration *c_SERS_* and the scattering volume around the Au nanoparticles. Only analyte molecules in the excitation volume, approximated by *A_laser_h_Au_*, can contribute to the signal. In a second step, the fraction of volume that is accessible to the analyte *f_Rh6G_* is taken into account. As a first approximation, we assume *f_Rh6G_* = 0.48 as taken from the unfilled volume in a cube filled by a sphere. Hereby, the number of contributing molecules is overestimated as this approximation does not represent the closest packing of the spheres, and as the hot spot volume is most probably smaller than the unoccupied space. Additionally, the contribution of molecules penetrating to the lower layers in the Au line is questionable. Analogously, we can give an estimate of the number of molecules contributing to the reference Raman signal by *N_Ref_ = c_Ref_ N_A_h_Gauss_A_laser_* with *h_Gauss_A_laser_* being the scattering volume. Taking the intensities of the peak at 766 cm^−1^, the EF was calculated to be 4.3 × 10^6^. This value is even higher than the values observed with printed nanoparticle SERS [15,19]. The calculation of the EF is presented in detail in the SI. Further optimization of the EF might be possible using different inks, or by combining the nanoparticle approach with a prestructured polymer substrate in order to create more sophisticated SERS structures. 

**Figure 6:**
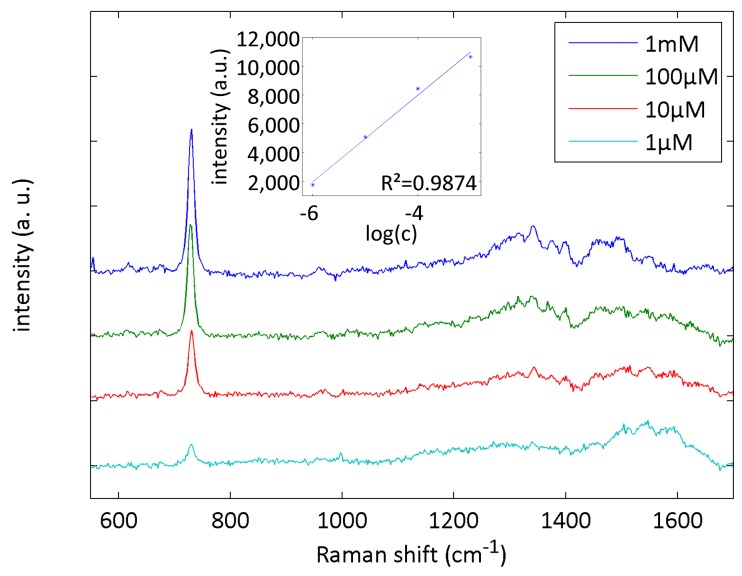
Microfluidic SERS measurements of adenosine solutions at concentrations from 1 µM and 1 mM. Acquisition time was 60 s and we averaged over four randomly chosen spots. The inset shows the linear relation between logarithmic concentration and intensity.

In order to prove their biomolecular relevance, the SERS chips were used for the SERS detection of adenosine, which is an important neuromodulator in the central nervous system [38,39]. Solutions with concentrations from 1 µM to 1 mM were pumped through the microfluidic chip. Due to the lower Raman cross-section of adenosine compared to Rh6G, we used an acquisition time of 60 s and averaged over four spots for each measurement. The results are shown in Figure 6. Again, a linear relationship between the intensity of the peak at 730 cm^−1^ can be given by I_730_ = 3002 × log(c) + 19,990. The detection limit of adenosine was calculated to 219 nM.

The quantitative SERS measurements with Rh6G and adenosine confirm the large potential of the fabricated microfluidic SERS chips for the quantitative detection of chemicals and biomolecules. 

## 4. Conclusions

We presented the fully R2R-compatible fabrication and evaluation of microfluidic chips for SERS analysis based on aerosol-jet-printed Au nanoparticles. We herewith addressed two main challenges of SERS, namely the low throughput and therefore the high cost of SERS structures and the integration into microfluidics for the investigation of solutions. We developed R2R hot embossing and R2R thermal bonding processes for the fabrication of microfluidic chips without the need for additional bonding layers. We optimized both processes and achieved full transfer of the microstructures in the embossing step and a strong bonding behavior of the microfluidic chips. Furthermore, the digital printing technology, aerosol jet printing, was exploited to generate highly-efficient and reproducible SERS-active areas in the microfluidic chips. We analyzed the reproducibility of the printing process concerning the SERS performance of the chips by comparing areas resulting from different tube temperatures and numbers of printing cycles. We achieved excellent results for the rather low tube temperature of 20 °C and a relatively high number of nine to 12 printing cycles, leading to spatially homogeneous nanoparticle layers with a high uniformity of SERS signals. The SERS enhancement factor was 4.3 × 10^6^ for Rh6G. Additionally, we have demonstrated the detection of the neuromodulator adenosine and thereby proved the suitability for bioanalytical applications. This work paves the way for fully R2R-fabricated, low-cost, microfluidic chips for biochemical SERS analysis.

## Supplementary Materials

The following are available online at http://www.mdpi.com/1424-8220/17/10/2401/s1, Figure S1: Master fabrication for R2R hot embossing, Figure S2: AFM measurements, Figure S3: Light microscope images of residues of NP ink, Figure S4: Influence of post treatments, Figure S5 dark field scattering spectra, Figure S6: SERS measurements for enhancement factor calculation.
