# Supplementary material for: Lab-on-Chip, Surface-Enhanced Raman Analysis by Aerosol Jet Printing and Roll-to-Roll Hot Embossing
Source: Sensors (Basel). 2017 Oct 20;17(10):2401. doi: 10.3390/s17102401 (PMC5677385; doi:10.3390/s17102401)
Supplement: Supplementary file 1 [file sensors-17-02401-s001.pdf]

sensors

MDPI

*Supplementary Information*

# Lab-on-Chip, Surface-Enhanced Raman Analysis by Aerosol Jet Printing and Roll-to-Roll Hot Embossing

**Anne Habermehl [1,*], Noah Strobel [1,2], Ralph Eckstein [1,2], Nico Bolse [1], Adrian Mertens [1], Gerardo Hernandez-Sosa [1,2], Carsten Eschenbaum [1,2,3] and Uli Lemmer [1,2,3]**

[1] Light Technology Institute, Karlsruhe Institute of Technology (KIT), Engesserstraße 13, 76131 Karlsruhe, Germany; noah.strobel@kit.edu (N.S.); ralph.eckstein@kit.edu (R.E.); nico.bolse@kit.edu (N.B.); adrian.mertens@kit.edu (A.M.); gerardo.sosa@kit.edu (G.H.-S.); carsten.eschenbaum@kit.edu (C.E.); uli.lemmer@kit.edu (U.L.)
[2] InnovationLab GmbH, Speyerer Straße 4, 69115 Heidelberg, Germany
[3] Institute of Microstructure Technology, Karlsruhe Institute of Technology (KIT), Hermann-von-Helmholtz-Platz 1, 76344 Eggenstein-Leopoldshafen, Germany
* Correspondence: anne.habermehl@kit.edu; Tel.: +49-721-608-44055

## 1. Master Fabrication for R2R Hot Embossing

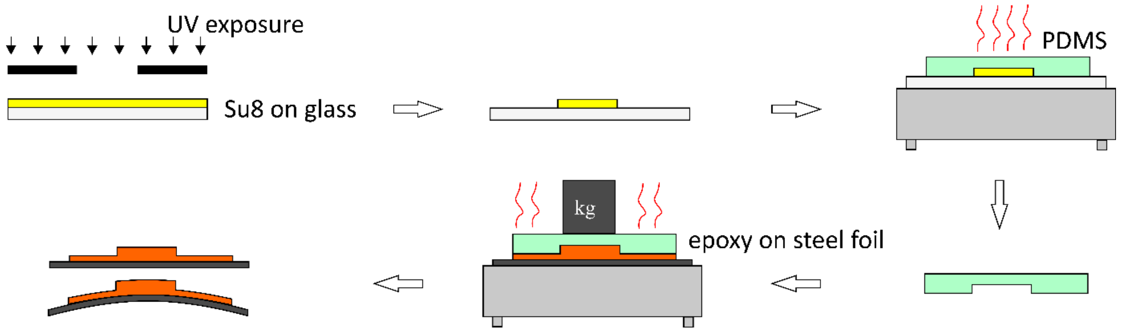


**Figure S1.** Replication processes for the fabrication of the epoxy master for roll-to-roll hot embossing.

## 2. AFM Measurements

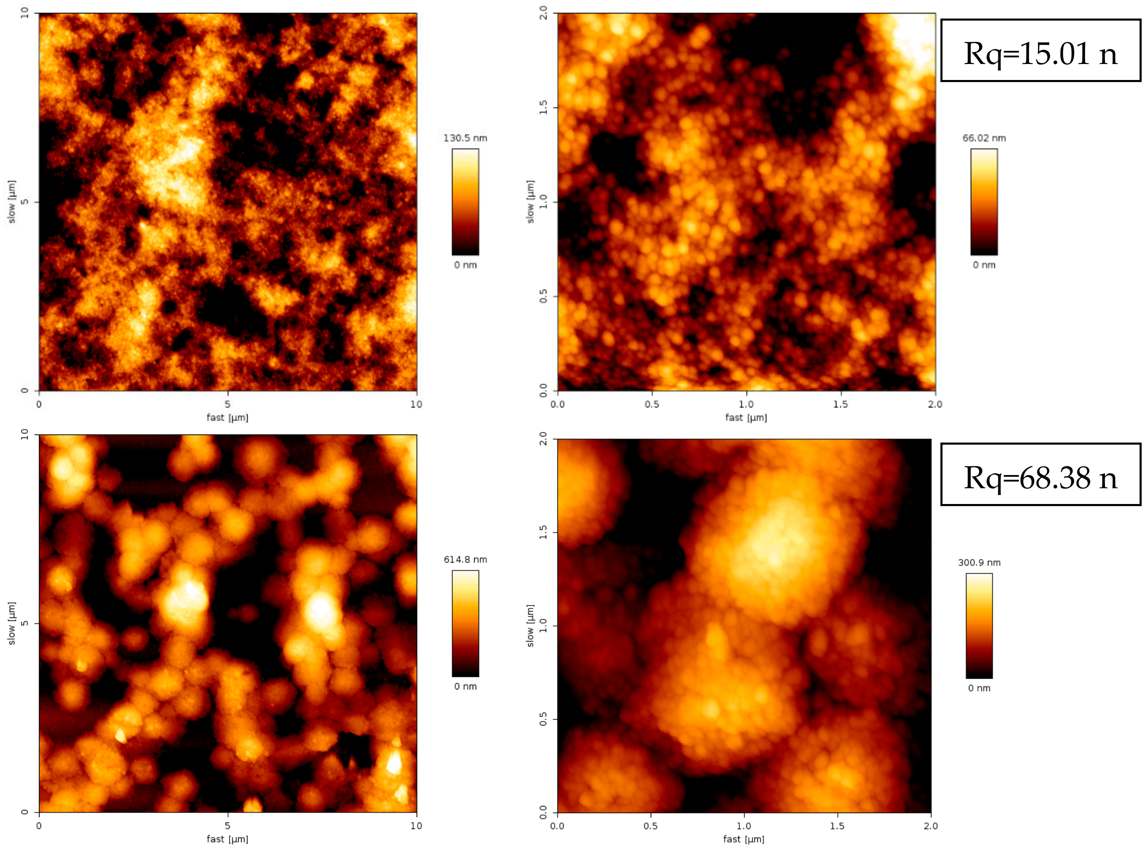


**Figure S2.** AFM analysis of nanoparticles printed with 20 °C (**up**) or 60 °C (**down**).

## 3. Residues of NP Ink

After the printing of the Au nanoparticle ink some residues are left on and between the nanoparticles (see Figure S3).

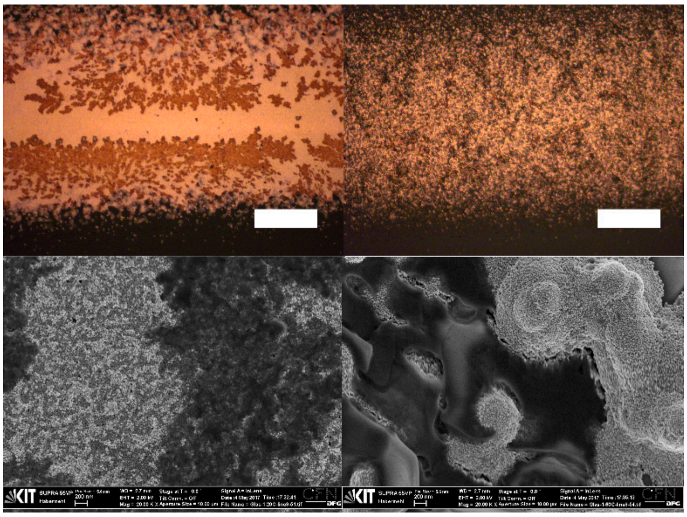

**Figure S3.** Light microscope and SEM images of residues on nanoparticle layers printed with 20 °C (**left**) and 60 °C (**right**).

We investigated different approaches to remove those residues. SEM-images of the resulting nanoparticle layers are given in Figure S4. Oxygen plasma treatment removes the residues but also reduces the enhancement of the Au structures, most probably due to the decreased volume of hot spots as the accessible area in between of nanoparticles (Figure S4c,d). Additionally, it prevents successful thermal bonding of the microfluidic chips. For both reasons oxygen plasma bonding is not

the approach of choice for the fabrication of microfluidic SERS. Vacuum treatment for 3 h results in a removal of the residues. Rinsing with water is the fastest and easiest possibility to remove the residues and can be easily integrated in the measurements protocol.

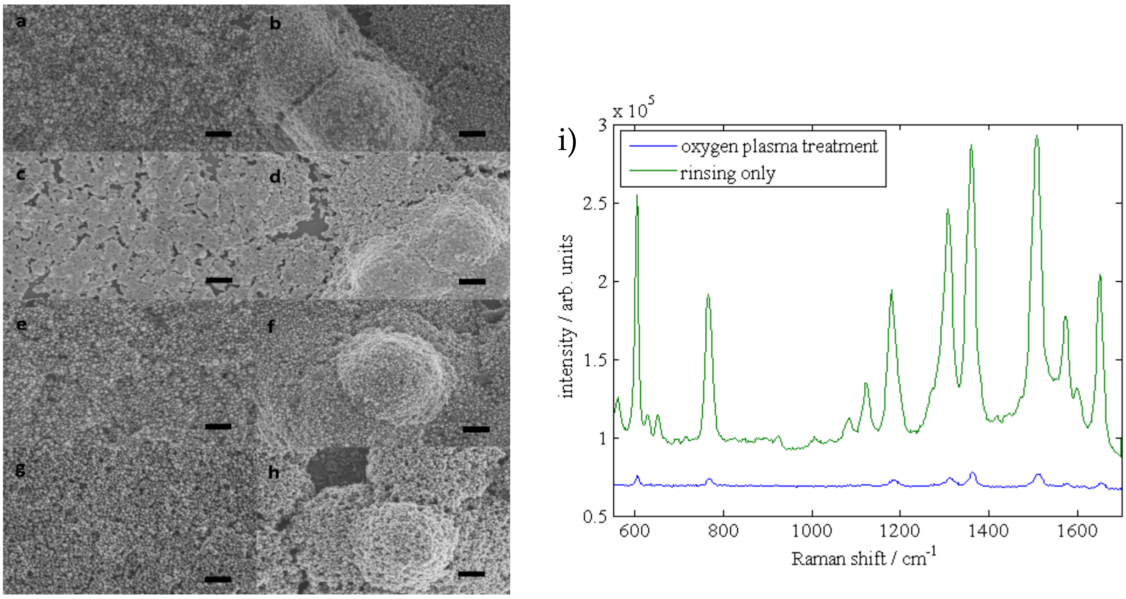


**Figure S4.** SEM images of printed nanoparticles without treatment (**a**,**b**), after 10 Min of oxygen plasma treatment (**c**,**d**), after storing in vacuum of $10^{-6}$ mbar for 3 h(**e**,**f**) and after rinsing (**g**,**h**), SERS spectra of 30 µL of 10 µM Rh6G solution dried on SERS substrates on an area of 20 × 20 $mm^2$ printed with 20 °C tube temperature, 10 s acquisition time (i).

## 4. Dark Field Scattering

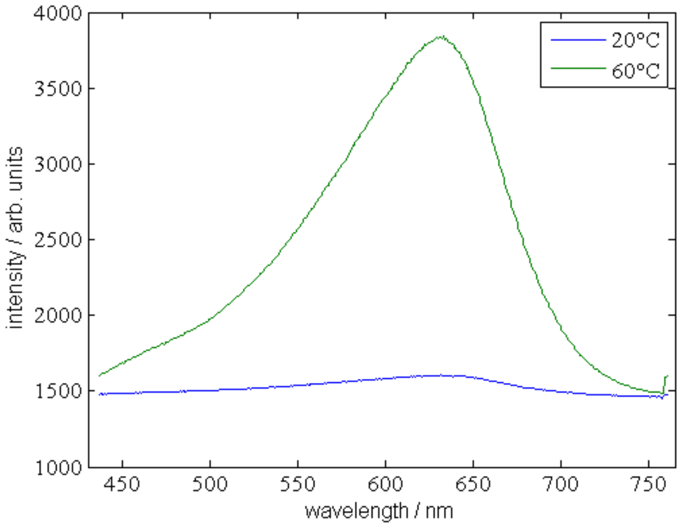


**Figure S5.** Dark field scattering spectra of 20 °C and 60 °C printed SERS substrates. Summed over a cross section of ~40 µm.

## 5. Enhancement Factor Calculation

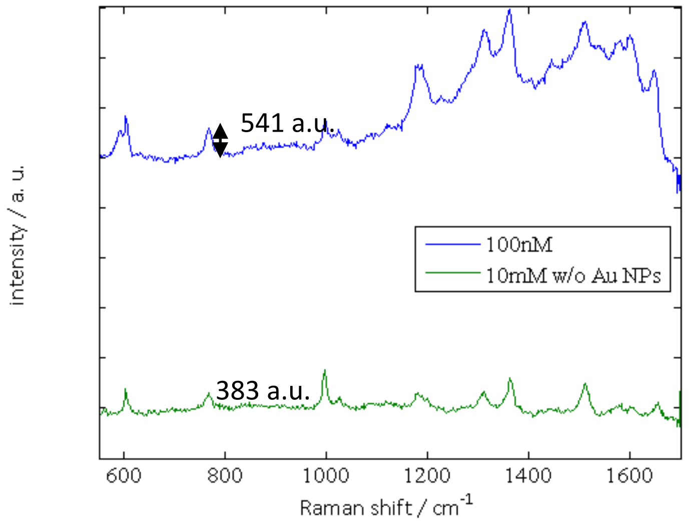


**Figure S6.** SERS measurement of 100 nM Rh6G solution, 0.9 mW, Raman reference of 10 mW with 2.6 mW; 10 s, 20 spots.

For the calculation of the enhancement factor we follow the well-known equation EF = $I_{SERS}/I_{ref} \times N_{SERS}/N_{ref}$. $I_{SERS}$ = 541 a.u. and $I_{ref}$ = 383 a.u. are taken from the measurements with 100 nM Rh6G solution on the Au nanoparticles and 10 mM Rh6G solution without nanoparticles (see Figure S6) and normalized for the excitation power used, which was 0.9 mW and 2.6 mW, respectively.

The number of molecules contributing to the SERS and Raman signal, respectively, can be calculated following $N_{SERS} = c_{SERS} N_A A_{Laser} h_{Au} f_{Rh6G}$ and $N_{Ref} = c_{Ref} N_A A_{Laser} h_{Gauss}$ with $A_{Laser} h_{Au}$ being the scattering volume around the Au nanoparticles with the thickness of the nanoparticle layer $h_{Au}$ and $A_{Laser} h_{Gauss}$ being the laser excitation volume, assuming a gaussian laser profile.

It follows EF = $I_{SERS,norm}/I_{ref,norm} \times (c_{Ref} h_{Gauss})/c_{SERS} h_{Au} f_{Rh6G}$.

With
$I_{SERS,norm}$ = 601.11 × $10^3$ a.u./W
$I_{ref,norm}$ = 147.31 × $10^3$ a.u./W
NA = 0.6
$c_{Ref}$ = 10 mM
$c_{SERS}$ = 100 nM
$h_{Gauss}$~5 µm
$h_{Au}$~1 µm
$f_{Rh6G}$ = 0.477 for a sphere in a cube, the enhancement factor can be calculated to be 4.3 × $10^6$.

© 2017 by the authors. Submitted for possible open access publication under the terms and conditions of the Creative Commons Attribution (CC BY) license (http://creativecommons.org/licenses/by/4.0/).
